# Detection of High-Risk Human Papillomavirus Genotypes Among HIV-Infected Women in Four States in Nigeria

**DOI:** 10.7759/cureus.57120

**Published:** 2024-03-28

**Authors:** Azuka P Okwuraiwe, Ebere L Ogbonne, Anthony O Adeniyi, Patrick I Ihurhe, Blessing O Musa, Temilade R Abe, Opeoluwa O Shodipe, Rosemary A Audu

**Affiliations:** 1 Genomics Strategic Core Platform, Medical Research Council Unit, Banjul, GMB; 2 Microbiology, Nigerian Institute of Medical Research, Yaba, NGA

**Keywords:** high-risk, hpv detection, hpv genotypes, hiv aids, human papillomavirus (hpv)

## Abstract

Introduction

The World Health Organization states that almost all cervical cancer cases are linked to infection with high-risk human papillomaviruses transmitted through sexual contact. Implementing effective surveillance and preventive measures would enable the prevention of most cervical cancer cases, especially in HIV-infected women. Every year, about 12,000 women in Nigeria are diagnosed, with almost 8,000 deaths. HPV cervical cancer testing capacity is low in Nigeria. Testing scale-up and sensitization efforts across health facilities, including cervical tissue sample collection, are needed to reduce the cases of cervical cancer. This study aimed to assess the genotype-specific prevalence of clinically relevant high-risk HPV among women living with HIV in Nigeria.

Methods

A descriptive, cross-sectional study was conducted among adult HIV-infected women attending health facilities in four Nigerian states. From August to October 2022, cervical tissue was collected into PCR cell media, transported to the Nigerian Institute of Medical Research, and assayed for HPV presence and genotype using the Cobas 6800 System (Roche Diagnostics). Statistical analysis was conducted with Stata 2.

Results

A total of 4423 cervical swab samples were tested. The ages of women ranged from 18 to 72 years (mean 36.61±8.61). In our study, we found that 16.3% of participants tested positive for HPV. Among the high-risk HPV genotypes detected, HPV16 was present in 1.44% of participants, HPV18 in 1.29%, and other high-risk HPV (OHR-HPV) in 11.35%. Additionally, co-infections were observed, with 0.98% of participants testing positive for both HPV16 and OHR-HPV, 1.12% for HPV18 and OHR-HPV, and 0.12% for HPV16, HPV18, and OHR-HPV concurrently. However, 7.4% of the total results were deemed invalid.

Conclusion

OHR-HPV is prevalent among HIV-infected women across the north and west geopolitical zones of Nigeria. Policies and interventions geared towards curtailing the incidence of cervical cancer are fervently solicited.

## Introduction

Human papillomavirus (HPV) infection is the most highly prevalent sexually transmitted infection, and genital HPV infections have a global prevalence of about 11-12% [1]. The highest prevalence of cervical HPV among women is in sub-Saharan Africa (24%), followed by Latin America and the Caribbean islands (16%), eastern Europe (14%), and South-East Asia (14%) [2]. Infection with HPV is known to be the major cause of cervical cancer [3]. Close to 90% of the general population will get infected with at least an HPV genital type at some point in their lives [4]. Those between the ages of 20 and 24 have the highest incidence of infection; however, no age is immune from acquiring HPV [5]. A significantly high number of new HPV infections in both sexes are described in Africa annually. Most genital HPV infections are asymptomatic, short-lived, and wane without causing disease [6]. It has been reported that every year about 12,000 women in Nigeria are diagnosed, and almost 8,000 die from the disease. There is low-level HPV cervical cancer testing in the country. Testing scale-up and sensitization efforts across health facilities, including cervical tissue sample collection, are needed to reduce the cases of cervical cancer.

HPV is a double-stranded DNA virus devoid of an envelope belonging to the *Papovaviridae* family. This family of viruses infects the cutaneous and mucosal epithelia. Almost 200 HPV genotypes have been identified, with more than 40 colonizing the genital tract [7]. All HPV infections are divided into two groups based on their carcinogenic properties, namely high-risk and low-risk. The high-risk HPV types comprise 16, 18, and 11 more genotypes. Others are classified as potential high-risk (which are 53, 66, 70, 73, and 82). The literature currently states that HPV16 and 18 are the most virulent high-risk genotypes, causing about 70% of all invasive cervical cancers in the world [8]. Genital HPV infections, with a global prevalence of about 11-12%, have been ranked as one of the most common viral agents of sexually transmitted infections worldwide [8].

The expected occurrence of HPV in distinctive areas of the world differs [9]. Compared to other regions, the highest prevalence of 22.1% is reported in Africa. Variations in the prevalence rates stated in different African countries exist, depending on the people being considered. In Sudan and Senegal, HPV prevalence has been reported to be 2.2% and 18%, respectively. Approximately 85% of deaths from cervical cancer occur in low- and middle-income countries, with a death rate 18 times that of wealthier countries [10]. There has been substantial evidence linking cervical cancer to sexually acquired HPV infection, particularly with HPV genotypes 16 and 18 [11].

Human immunodeficiency virus (HIV) infection is also a strong determinant of cervical cancer [12]. Compared to women in the general population, those living with HIV are at higher risk of incidental and persistent HPV infection. This is because HIV weakens the immune system, making individuals more susceptible to acquiring HPV and developing persistent infections with high-risk HPV strains [13]. Both HIV and HPV infections share common risk factors, including unprotected sexual activity, multiple sexual partners, and engaging in high-risk sexual behaviors such as anal sex. Individuals who are at risk for HIV infection are also often at risk for HPV infection, and vice versa. Coinfection with HIV and HPV is common due to shared routes of transmission [13].

HIV-infected individuals are at increased risk for HPV-related diseases, including genital warts, cervical dysplasia, cervical cancer, anal cancer, and other HPV-related malignancies [14]. HIV infection can affect the natural history and progression of HPV-related diseases. HIV-positive individuals with HPV infection are more likely to develop high-grade cervical dysplasia and invasive cervical cancer compared to HIV-negative individuals. The presence of HIV infection can complicate the screening, diagnosis, and management of HPV-related diseases. HIV-positive individuals may have higher rates of HPV persistence, recurrence, and treatment failure, necessitating more frequent and intensive screening, surveillance, and treatment strategies [14].

Antiretroviral therapy (ART) has been shown to improve immune function and reduce the risk of HPV-related diseases in HIV-positive individuals. However, the effectiveness of ART in preventing and treating HPV-related diseases may vary depending on factors such as CD4 cell count, HIV viral load, ART adherence, and HPV genotype [15].

Addressing the dual burden of HIV and HPV infections requires integrated approaches to prevention, screening, and treatment. Furthermore, women living with HIV (WLHIV) are more likely to harbor several oncogenic genotypes and are thereby prone to developing cervical precancerous or cancerous lesions [15]. Over the last two decades, the scaling-up of antiretroviral therapy (ART) has dramatically increased the life expectancy of WLHIV [16], leading to a higher incidence and prevalence of cervical cancers related to HR-HPV [17,18]. This study aimed to determine the prevalence of genital HPV infection and genotypes among HIV-positive women in four states of Nigeria.

## Materials and methods

Ethical considerations

Ethical approval for this study was obtained from the Nigerian Institute of Medical Research (NIMR), and signed informed consent was obtained from all participants recruited for this investigation. Participation was entirely voluntary, and the handling of subjects conformed to the Declaration of Helsinki.

Inclusion and exclusion criteria

The inclusion criteria included sexually active HIV-infected women of Nigerian origin, 18 years of age and older, having no co-infections, and having consented to the study. The exclusion criteria included pregnant women, persons under 18 years, HIV-hepatitis B and C co-infected persons, HIV-tuberculosis co-infected persons, those already having cervical cancer, and those who declined to give consent to the study.

Study design

This was a cross-sectional cohort study of HIV patients who received treatment at HIV reference clinics across 65 health facilities in southwest and northern Nigeria. Patient recruitment was carried out between August and October 2022.

Sample population

High-swab cervical samples were collected from 4423 HIV-infected persons from various healthcare facilities in North and West Nigeria. During patients’ routine visits to their health facilities, HPV surveillance was incorporated into their HIV management and care. Adult HIV-positive women, aged 18 to 72, who assessed clinical care in health facilities in four states from different geopolitical zones (Lagos in the south-west, Niger in the north-central, Kebbi in the north-east, and Kaduna in the north-west) in Nigeria were enrolled.

Data collection

A pre-tested simple questionnaire was administered by health workers at the various facilities in the four study states to the participants to ascertain their age, how long they had been diagnosed with HIV infection, and other demographics.

Sample collection and analysis

Medical personnel subsequently obtained cervical tissue samples with sterile cytobrushes into PCR cell media and stored them at room temperature (25 to 32°C). Sample collection was carried out between August and October 2022. These were transported at room temperature in bulk to the Center for Human Virology and Genomics (CHVG) of the NIMR.

After sample verification, one milliliter of each sample was manually pipetted into a 4 ml (75 × 11mm) rohren tube (Sarstedt, Germany) using 1000 µl filtered sterile pipette tips. An assay for HPV detection and genotype was subsequently performed on the automated Cobas 6800 System, manufactured by Roche Diagnostics (Mannheim, Baden-Württemberg, Germany). The sample tubes placed on racks were manually loaded onto the sample module of the Cobas 6800 analyzer, scanned, accepted, and automatically moved to the transfer (second) module. Here, samples were pipetted into processing plates, which were then transferred into the extraction (third) module for automated sample extraction to obtain HPV DNA. Isolated DNA was transferred into 96-well microwell plates and moved to the amplification/detection (fourth) module.

The process takes approximately four hours, and results are displayed and printed afterward. Results were subsequently entered into request forms, verified by correlating sample request forms with lab result sheets, signed, and dispatched by a logistics delivery company to the various health facilities, and filed for patients’ consultation with doctors.

Statistical analysis

Patient data was collected on request forms and entered into Microsoft Excel 2016 (Microsoft Corporation, Redmond, WA, USA) for cleaning. Statistical analysis was conducted with Stata 2 (StataCorp LLC, Texas, USA). The parameters utilized were mean, standard deviation, and frequency distribution as percentages.

## Results

A total of 4423 cervical swab samples were collected from the four states and tested, with 4095 (92.6%) results being valid. The age of the women ranged from 18 to 72 years, with a mean (±SD) of 36.61 (±8.61) years. The outcome of the testing is shown in Table 1. Excluding the invalid results, the highest HPV DNA positivity was observed in Lagos State (20.1%), followed by Kaduna State (19.5%). Niger and Kebbi states trailed with HPV DNA positivity of 14.4% and 14.3%, respectively. ﻿

**Table 1:** HPV DNA testing results across four states in Nigeria: positive, negative, and invalid cases Invalids are HPV-negative specimens with a negative beta-globulin (internal control) result. They are flagged as invalid, preventing the reporting false negatives.

Nigerian State	Tested (%)	Positive (%)	Negative (%)	Invalid (%)
Lagos	481 (10.9)	90 (18.7)	357 (74.2)	34 (7.1)
Niger	1543 (34.9)	206 (13.3)	1228 (79.6)	109 (7.1)
Kaduna	1147 (25.9)	208 (18.1)	859 (74.9)	80 (7.0)
Kebbi	1252 (28.3)	164 (13.1)	983 (78.5)	105 (8.4)
Total	4423 (100)	668 (15.1)	3427 (77.5)	328 (7.4)

The prevalence of high-risk HPV in the study was 16.3%, and the genotypes of HPV obtained were HPV16 1.44%, HPV18 1.29%, and other high-risk (OHR) HPV 11.35%, amongst others, as shown in Table 2. The distribution of the different HPV genotypes across the Nigerian states investigated indicates OHR as the most observed genotype, as shown in Table 3. 

**Table 2:** Prevalence of HPV genotypes 16, 18, and other high-risk types in HIV-positive women in Nigeria: a descriptive analysis The percentages are calculated with respect to the total number of HPV-positive cases obtained, i.e., N = 668.

HPV genotype	Number (%)
16	59 (1.44)
18	53 (1.29)
OHR	465 (11.35)
16 & OHR	40 (0.98)
18 & OHR	46 (1.12)
16, 18 & OHR	5 (0.12)

**Table 3:** HPV genotype distribution: prevalence of types 16, 18, and other high-risk (OHR) in women across four Nigerian states

Nigerian State	HPV Pos (%)	OHR HPV(%)	HPV 16 (%)	HPV 18 (%)	HPV 16 & OHR (%)	HPV 18 & OHR (%)	HPV 16,18 & OHR (%)
Lagos	90 (18.7)	69 (76.6)	11 (12.2)	7 (7.7)	2 (2.2)	1 (1.1)	0 (0)
Niger	206 (13.3)	154 (74.7)	16 (7.7)	19 (9.2)	9 (4.4)	8 (8.2)	0 (0)
Kaduna	208 (18.1)	135 (64.9)	17 (8.2)	15 (7.2)	13 (6.3)	25 (12)	3 (1.4)
Kebbi	164 (13.1)	107 (65.2)	15 (9.1)	12 (7.3)	16 (9.7)	12 (7.3)	2 (1.2)
Total	668 (15.1)	465 (69.6)	59 (8.9)	53 (7.9)	40 (5.9)	46 (6.9)	5 (0.7)

## Discussion

Cervical cancer has continued to be a silent scourge, affecting women in their prime years across the world, Nigeria included. The Federal Government of Nigeria (FGN), through the FMoH, therefore decided to implement a program to screen women for HPV, the major causative agent of cervical cancer. To support the FGN in implementing its national cervical cancer control strategic plan, it reached out to key stakeholders to engage in a partnership to maximize existing resources and efforts to catalyze the government’s response toward reducing cervical cancer occurrence.

The focus was on secondary cervical cancer prevention while strengthening linkages to care and patient tracking mechanisms. One of the objectives was to improve the screening of WLHIV with HPV DNA testing, which is regarded as the gold standard for cervical cancer screening as they are highly predisposed to developing pre-cancer [18]. The results obtained indicate a high prevalence of HPV among this group of women. There was an overall HPV prevalence of 16.3% in the HIV population studied. However, the prevalence of HPV 16 and 18 were minor at 1.44% and 1.29%, respectively, with a majority (11.35%) of HPV detected being found to be in the 'other high risk' category. This discovery is similar to that found in a Romanian study of HPV-co-infected HIV women, where the most frequent genotypes were 31 (42.1%), 56 (31.57%), and 53 (15.78%) [19]. It is a bigger challenge for such co-infected women in society who are already battling with chronic HIV disease. The risk of HPV infection in HIV patients has been associated with an impaired immune response to HPV [20]. As the results portray, OHR-HPV was prevalent across Nigeria's north and southwest geopolitical zones. Regrettably, the occurrence of stage 4 cervical cancer is higher among women of African origin as compared to Caucasian women [20], with its treatment costing about $56,000 annually [21]. In this study, follow-up care was implemented for patients who were HPV-positive. They were triaged and treated by thermal ablation and the loop electrosurgical excision procedure (LEEP). The number of invalid results obtained was 328 (7.4%), which is high. The invalid results obtained were likely due to improper cervical tissue sample collection.

A significant proportion of cervical cancers in Nigerian women can be prevented if strategies such as early HPV DNA-based screening and HPV vaccination are made more accessible and implementable. HPV is an important cause of cervical cancer, but it is not a sufficient cause. Other cofactors are important for the development of HPV cervical cancer. Tobacco smoking, excessive parity, long-term hormonal contraceptive use, and co-infection with HIV have been recognized as cofactors [22]. Co-infection with chlamydia trachomatis and herpes simplex virus type-2, immunosuppression, and positive nutritional deficiencies are possible cofactors. However, these cofactors were not measured in this study. Immunological, genetic, host and viral factors such as variant type and viral load may also be important but have not been identified.

Study limitations

The complete demographic data of all participants could not be obtained as some facilities had challenges transmitting the information. Secondly, co-factors like smoking, contraceptives, and others stated above were not part of the data collected.

Study implications

The study highlights the prevalence of HPV in an already burdened group of women and the risk of cervical cancer in that population if adequate early intervention is not carried out. Future research would need to monitor the success of interventions and identify areas for improvement.

## Conclusions

This study presents novel findings regarding the distribution of HPV genotypes among HIV-infected women in specific regions of Nigerian communities. Notably, HPV prevalence is high among HIV-positive women. Of particular concern is the predominance of genotypes distinct from the commonly known oncogenic types, HPV 16 and 18. Consequently, there is a pressing need for the development of new national policies and interventions. These may include strategies such as the utilization of immune-enhancing topical treatments, the implementation of observational and palliative medical approaches to manage high-grade lesions, and the deployment of techniques aimed at preventing the progression of cervical cancer.
